# Design, Characterization, and Liftoff of an Insect-Scale Soft Robotic Dragonfly Powered by Dielectric Elastomer Actuators

**DOI:** 10.3390/mi13071136

**Published:** 2022-07-18

**Authors:** Yufeng Chen, Cathleen Arase, Zhijian Ren, Pakpong Chirarattananon

**Affiliations:** 1 Research Laboratory of Electronics, Department of Electrical Engineering and Computer Science, Massachusetts Institute of Technology (MIT), Cambridge, MA 02139, USA; carase@mit.edu (C.A.); zhijianr@mit.edu (Z.R.); 2 Department of Biomedical Engineering, City University of Hong Kong, Hong Kong, China

**Keywords:** biologically inspired robot, soft robot, dielectric elastomer actuator, flapping-wing, micro-aerial-vehicle

## Abstract

Dragonflies are agile and efficient flyers that use two pairs of wings for demonstrating exquisite aerial maneuvers. Compared to two-winged insects such as bees or flies, dragonflies leverage forewing and hindwing interactions for achieving higher efficiency and net lift. Here we develop the first at-scale dragonfly-like robot and investigate the influence of flapping-wing kinematics on net lift force production. Our 317 mg robot is driven by two independent dielectric elastomer actuators that flap four wings at 350 Hz. We extract the robot flapping-wing kinematics using a high-speed camera, and further measure the robot lift forces at different operating frequencies, voltage amplitudes, and phases between the forewings and hindwings. Our robot achieves a maximum lift-to-weight ratio of 1.49, and its net lift force increases by 19% when the forewings and hindwings flap in-phase compared to out-of-phase flapping. These at-scale experiments demonstrate that forewing–hindwing interaction can significantly influence lift force production and aerodynamic efficiency of flapping-wing robots with passive wing pitch designs. Our results could further enable future experiments to achieve feedback-controlled flights.

## 1. Introduction

Aerial insects have demonstrated remarkable flight capabilities owing to unique scaling advantages. Due to the diminishing inertial effects, insects are resilient against strong in-flight collisions [1] that are otherwise challenging for larger-scale birds and drones. In addition, insects can generate large aerodynamic forces and torques compared to their inertia, which leads to elusive flight maneuvers such as fast turning [2] and quick recovery in response to external disturbances [3]. This combination of mechanical resilience and flight agility enables insects to navigate in highly cluttered environments and interact with delicate objects. For instance, bees can collectively pollinate flowers and construct hives, and these tasks remain challenging for larger-scale aerial robots.

Motivated by the prospect of these potential applications, there has been a growing interest in developing insect-scale aerial robots over the past decade. Commercially available motors are unsuitable for constructing sub-gram aerial robots because the power density of motors quickly diminishes while friction increases at the millimeter scale. Wood et al. [4] developed microscale (10–200 mg) piezoelectric actuators that meet the stringent power requirement on achieving flight. In addition, the team invented the Smart Composite Manufacturing (SCM) method [5] that leads to a novel class of flexure-based microscale (0.1–20 mg) transmissions and structural components. The team created a class of sub-gram aerial robots named “RoboBees”, and they demonstrated feedback-controlled hovering flight [6], sensing autonomy [7], perching on compliant objects [8], hybrid aerial–aquatic locomotion [9], and solar-powered takeoffs [10]. Several of these demonstrations take advantage of the robots’ small scale and they are challenging for the state-of-the-art larger-scale aerial robots. In addition, other types of microscale actuators (e.g., electromagnetic actuator) [11] and propulsion methods (e.g., electrohydrodynamic propulsion) [12] were developed in parallel to demonstrate liftoff of sub-gram aerial robots.

**Figure 1:**
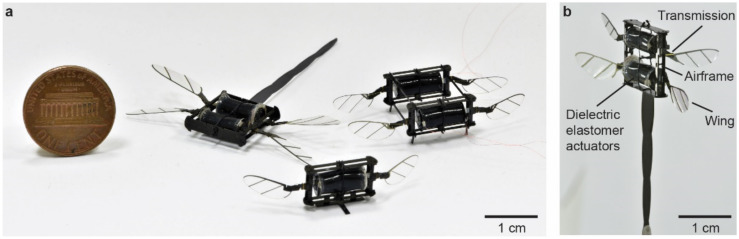
Illustration of insect-scale flapping-wing robots. (**a**) An image showing three insect-scale flapping-wing robots powered by DEAs. In this work, we design and characterize a 317 mg dragonfly-like (left) flapping-wing robot and investigate forewing–hindwing interactions. (**b**) The robot consists of two DEAs, four wings, an airframe, and four sets of transmissions and wing hinges.

In recent years, we created power-dense dielectric elastomer actuators (DEAs) and incorporated them into a new class of insect-scale soft aerial robots [13,14,15] (Figure 1a). These soft-actuated aerial robots have demonstrated insect-like resilience [14] and agility [13]. However, despite the improvement in the DEA design and fabrication, these soft actuators require over 500 V [15] to operate, which imposes significant challenges for developing compact and efficient high-voltage power electronics. To achieve power autonomous flight in future soft aerial robots, it is important to improve a robot’s aerodynamic efficiency and net lift.

Existing microscale flapping-wing robots take inspirations from *Diptera* flies that use a pair of wings for generating lift forces. In contrast, dragonflies (*Odonata*) use two pairs of wings during flight, and they change the phase between forewings and hindwings during hovering flight and takeoff [16]. The aerodynamic interaction between forewings and hindwings can influence aerodynamic efficiency, net lift production, and robot body oscillations during flight. Leveraging this interaction, a dragonfly can demonstrate exquisite body-flip maneuvers [17,18,19,20] through modulating its flapping-wing kinematics. Investigating forewing–hindwing interactions can benefit the efficiency and agility of flapping-wing robots. Several dragonfly-like robots were developed in previous studies [21,22] to investigate dragonfly flight. However, existing dragonfly-like robots are substantially heavier than the insects, making it difficult to use them for aerodynamic studies. An at-scale dragonfly robot would be beneficial to match the Reynolds number and study at-scale fluid–structure interactions.

In this work, we develop the first at-scale robotic dragonfly (robot on the left in Figure 1a) and experimentally study forewing–hindwing interactions. The robot (Figure 1b) consists of a carbon fiber airframe, two DEAs that can independently drive forewings and hindwings, four-bar transmissions, and wing hinges. We drive the DEAs at different voltages, frequencies, and relative phases and measure the resulting flapping kinematics and lift forces. Our experiments show that in-phase flapping can increase the net lift force by 19% compared to out-of-phase flapping, and this phenomenon relates to the forewing–hindwing interactions caused by downwash [23,24]. Under the maximum driving condition, the robot generates 4.64 mN of net lift force, corresponding to 1.49 times of the robot weight. Our result shows that at-scale flapping-wing robots can leverage forewing–hindwing interactions to enhance lift production, and this new robot platform will further benefit future works on demonstrating feedback-controlled aerial maneuvers.

## 2. Materials and Methods

### 2.1. Design of the Soft Robotic Dragonfly

**Figure 2:**
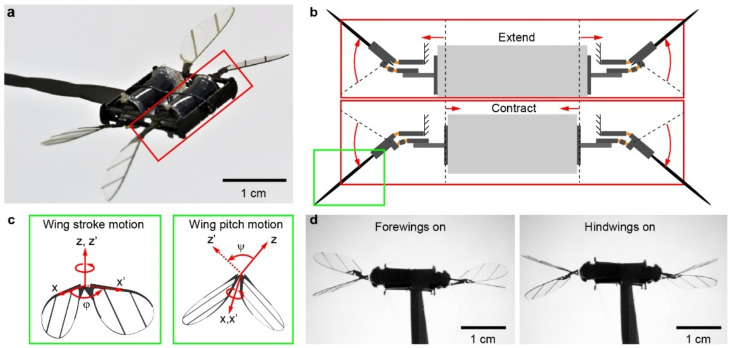
Design of an at-scale soft robotic dragonfly. (**a**) Perspective view of the 317 mg robot. The robot has two DEAs and each independently drives a pair of wings. (**b**) A DEA extends or contracts in response to a high-voltage driving signal. The two ends of the DEA connect to linear four-bar transmissions, which converts the DEA’s translational motion into the rotational wing stroke motion. (**b**) corresponds to the red inset shown in (**a**). (**c**) The DEA directly controls the wing stroke motion (ϕ), and the wing pitch rotation (ψ) is passively mediated by a compliant wing hinge. (**c**) corresponds to the green inset shown in (**b**). (**d**) The two DEAs can be actuated independently. The two high-speed camera images show experiments in which either the forewings (**left**) or the hindwings (**right**) are turned on.

In this section, we describe the design and fabrication of the soft robotic dragonfly. In prior works [13,14,15], we developed a soft aerial robot in which a pair of wings is driven by a single DEA. To investigate forewing–hindwing interactions, we create a new design in which two DEAs are positioned in series. Figure 2a shows this dragonfly-like robot where each DEA drives a pair of wings. The length and diameter of the DEAs are 9 mm and 5 mm, respectively. In this work, all four wings have the identical shape and inertia. The wingspan and mean chord length are 10 mm and 3.3 mm, respectively. The forewings and hindwings are separated by half of the mean chord length (1.6 mm), allowing strong mutual interactions. Our robot weighs 317 mg, which is similar to that of real dragonflies in nature. Our fabrication method [14] can construct aerial robots whose size and weight are in the range of 2–10 cm, 0.05–1 g.

The DEAs are made using an existing fabrication method [14]. They consist of alternating elastomer (Elastosil P7670, Wacker, Munich, Germany) and electrode (NanoC, Invisicon 3500, Westwood, MA, USA) layers and operate in the range of 1300 to 1700 V. A high-frequency (300–400 Hz) sinusoidal driving voltage causes the DEA to elongate and contract due to the electrostatic stress. The two ends of the DEAs are connected to linear four-bar transmissions, which convert a DEA’s linear translational motion to the robot’s wing stroke motion. Figure 2b illustrates this actuation design. The robot wings sweep forward when the DEA extends, and they sweep backward when the DEA contracts. This back-and-forth oscillation corresponds to the wing stroke motion (ϕ), which is fully controlled by the DEA (Figure 2c). To generate lift forces, the flapping-wing motion needs to have at least two degrees of freedom. In our design, the wing pitch motion $\left(\psi \right)$ is passively mediated by a compliant wing hinge (Figure 2c).

While this underactuated design simplifies robot fabrication, it still enhances forewing–hindwing interactions because the passive wing pitch motion is influenced by the induced flow from the forewings. In this work, we focus on studying how forewing and hindwing interactions impact flapping kinematics and the associated forces. Since each DEA drives two wings, the left and right wings are tightly coupled. In contrast, the two DEAs can drive the set of forewings and hindwings independently. Figure 2d shows flapping demonstrations in which only forewings (left panel of Figure 2d) or hindwings (right panel of Figure 2d) are turned on, respectively.

### 2.2. Experimental Setup

We conduct two types of at-scale experiments: static flapping and liftoff experiments. They allow us to measure the robot flapping kinematics and the associated forces at an operating condition. Here the robot driving condition refers to a combination of voltage amplitude, frequency, and phase for each DEA. In this work, the driving voltage amplitude and frequency of the two DEAs are set to the same values. The relative phase between the two DEAs is another input parameter. To measure the flapping kinematics and the lift force, we drive the robot twice at the same operating condition and use two different setups for measuring these two quantities. In the next two subsections, we describe the experimental setup for each test.

#### 2.2.1. Static Flapping-Wing Experiments

**Figure 3:**
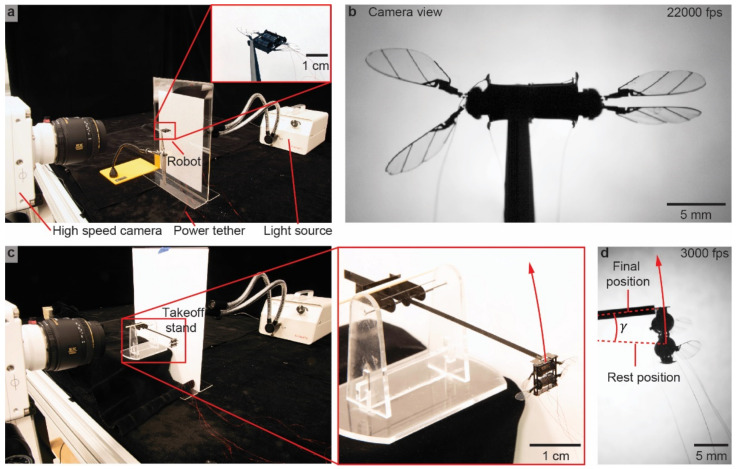
Experimental setup for measuring the robot flapping kinematics and mean lift force. (**a**) Setup of a static flapping-wing experiment. A backlight illuminates the robot, and the flapping-wing motion is captured by a high-speed camera at 22,000 fps. (**b**) A sample image that is captured by the high-speed camera when the robot operates at 350 Hz. (**c**) Setup of robot liftoff experiments. The red inset shows a robot is installed on the liftoff test stand. (**d**) A sample image that is captured by the high-speed camera during robot liftoff.

We conduct static flapping-wing experiments to measure the wing motion. Figure 3a shows an image of the experimental setup. The robot is affixed with a reverse-action tweezer. A halogen light source (Amscope HL150-A, AmScope, Irvine, CA, USA) illuminates the robot from the back. The light passes through a diffuser and shines into a high-speed camera (Phantom VEO 710, Vision Research Inc., Charlottetown, PE, Canada). The robot is driven by two DEAs, which are independently controlled by two high-voltage amplifiers (Trek 2220, Trek, Inc., New York, NY, USA). We wrote a custom Simulink control software that drives the amplifiers. The input driving signals are given by:

(1) $$\begin{array}{c} v_{1}\left(t\right)=V_{amp}+V_{amp}\cos\left(2\pi ft\right)\\ v_{2}\left(t\right)=V_{amp}+V_{amp}\cos\left(2\pi ft+\theta \right) \end{array}$$

Here the $v_{1}\left(t\right)$ and $v_{2}\left(t\right)$ are the instantaneous driving voltages for the two DEAs, $V_{amp}$ is the voltage amplitude, f is the driving frequency, and θ is the phase shift between the two DEAs. In this work, the driving parameters $V_{amp}$, f, and θ are in the range of 1450 to 1600 V, 320 to 410 Hz, and 0 to 180 degrees, respectively.

To drive the DEAs and sample the flapping kinematics with a high temporal resolution, our control hardware and high-speed camera run at frequencies of 20 kHz and 22 kHz, respectively. This implementation ensures each flapping-wing cycle has over 50 sample points. Figure 3b shows a sample high-speed camera image. The stroke and pitch kinematics can be extracted through tracking key wing features [15] in each frame of a video. This sample image shows the left- and right-wing kinematics are not symmetric due to imprecision in the robot assembly process. Supplementary Video S1 shows two flapping-wing experiments.

#### 2.2.2. Liftoff Experiments and Lift Force Measurement

In addition to quantifying the robot flapping-wing kinematics at an operating condition, we further measure the robot lift force using a custom setup. Figure 3c shows the robot mounted on a takeoff stand. This stand is balanced around a low-friction pivot point. If the robot can generate a net lift force that exceeds its weight, then the robot can accelerate upward (inset of Figure 3c). Figure 3d shows an image taken from a liftoff experiment. Supplementary Video S2 compares two liftoff experiments. We drive the robot for a fixed time Δt and measure its ascent angle γ. Based on these measurements and other robot parameters, we can calculate the time-averaged lift force.

The equation of motion is given by:

(2) $$I\ddot{\gamma }=(F_{L}-mg\cos\gamma )L$$

where $F_{L}$ is the time-averaged lift force, m is the sum of the robot mass and the payload, L is the distance between the robot and the pivot point, and I is the total moment of inertia contributed by the robot, the balance beam, and the payload, calculated with respect to the rotational axis. We choose a large moment arm (L= 9.2 cm) and a small operating time (Δt= 0.15 s) such that the final rotation angle is small ($\gamma_{f}<10{}^{\circ}$). This leads to the approximation that cosγ≈1, which simplifies the solution of Equation (2):

(3) $$\gamma_{f}=\frac{1}{2}\ddot{\gamma }\Delta t^{2}=\frac{1}{2I}\Delta t^{2}\left(F_{L}-mg\right)L$$

In a liftoff experiment, we can measure the final rotation angle $\gamma_{f}$ from the high-speed video and back calculate the net lift force:

(4) $$F_{L}=\frac{2I\gamma_{f}}{\Delta t^{2}L}+mg\;$$

The validity of Equation (4) can be verified during the calibration experiments. When subject to the same driving signal, the lift force $F_{L}$ is anticipated to remain unchanged. Therefore, the final rotation angle $\gamma_{f}$ should depend on the mass of the payload included in the term m.

## 3. Results

In this section, we investigate the influences of three kinematic parameters $\theta ,\;f,\text{ and }V_{amp}$ on robot lift force production. After the calibration for the liftoff tests, we measure the robot wing motion and lift forces as these driving parameters vary. The detailed results and analysis are described in the following sections.

### 3.1. Calibration for Liftoff Tests

**Figure 4:**
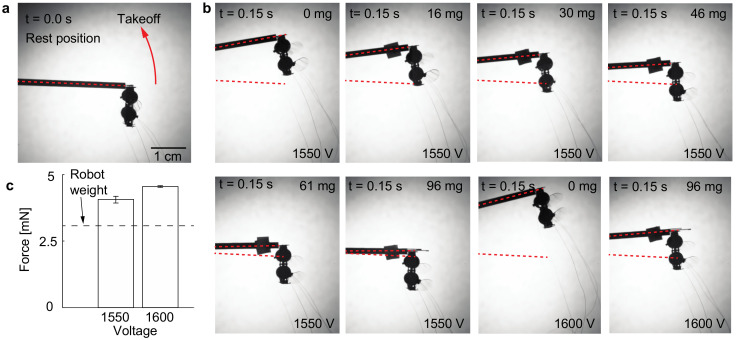
Calibration of mean lift force measurement. (**a**) The robot rest position at t = 0.0 s. (**b**) Positions of the robot at t = 0.15 s. The robot carries different payloads in these tests and it is either operated at 1550 V or 1600 V. (**c**) Measured mean lift forces at the two driving conditions. The error bars indicate the minimum and maximum measurements.

Prior to driving the robots at different operating conditions, we conduct a sequence of calibration liftoff tests and quantify the accuracy of our design. Figure 4 shows a sequence of liftoff experiments in which we repeatedly drive the robot at the same condition and place different payloads on the robot. Figure 4a shows the robot at its rest position. We drive both DEAs in phase with a 1550 V and 350 Hz sinusoidal input while varying the robot payload. The first six panels in Figure 4b show the robot’s positions after 0.15 s of operation. The robot cannot lift off when the payload exceeds 100 mg. We also perform two additional experiments that increase the driving voltage to 1600 V. The last two panels in Figure 4b compare the robot liftoff positions before and after a 96 mg payload is added to the robot. Using Equation (4), we calculate the net lift force for each of these liftoff experiments with the measured angle $\gamma_{f}$. To account for the added payload, we update the values of net inertia I and mass m accordingly. Figure 4c shows the calculated lift forces. For the six experiments where the robot is driven at 1550 V, the average force is 4.1 mN. The error bars indicate that the minimum and maximum measured values are within 4% of the mean value. For the two experiments driven at 1600 V, the robot lift increases to 4.6 mN with a discrepancy of 1%. The dotted lines in Figure 4c show the robot weight. At these test driving conditions, the robot achieves a lift-to-weight ratio of 1.31 and 1.49, respectively. These liftoff tests show our net lift measurement is accurate within 5% and our robot can generate sufficient forces to take off. In the next experiments, we use this liftoff setup for measuring the robot lift forces at different operating conditions.

### 3.2. Influence of Relative Phase θ on Lift Force Production and Wing Kinematics

**Figure 5:**
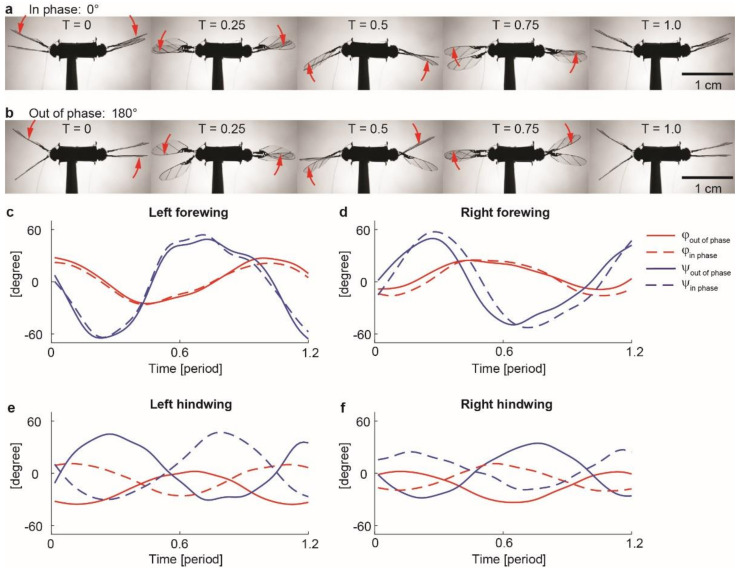
Comparison of in-phase and out-of-phase flapping kinematics. (**a**,**b**) Image sequences that show one period of in-phase (**a**) and out-of-phase (**b**) flapping experiments. The red arrows indicate the direction of wing stroke motion. (**c–f**) Tracked wing stroke (ϕ) and pitch (ψ) kinematics of the experiments shown in (**a**,**b**).

In the first set of flapping-wing experiments, we drive both DEAs at 1550 V and 350 Hz while varying the phase θ in range of 0° to 180°. Here 0° and 180° denote in-phase and out-of-phase flapping motion, respectively. Figure 5a and b show image sequences of the robot flapping kinematics. In the in-phase flapping experiment (Figure 5a), the forewings and hindwings have synchronous wing stroke motion. In contrast, in the out-of-phase flapping experiment (Figure 5b), the forewings and hindwings have opposing wing stroke motion. Supplementary Video S1 compares the in-phase and out-of-phase flapping experiments.

Based on these high-speed videos, we manually extract the time-varying flapping kinematics. Figure 5c–f show the tracked stroke (ϕ) and pitch $\left(\psi \right)$ kinematics for each wing. In these plots, the red and blue lines represent wing stroke (ϕ) and pitch $\left(\psi \right)$ motion, respectively. The solid and dotted lines compare the in-phase and out-of-phase experiment. We observe that while the forewing kinematics are similar (Figure 5c,d), the hindwing kinematics are 180° out of phase (Figure 5e,f). Furthermore, the wing stroke motion (red) is approximately sinusoidal because it is directly commanded by a sinusoidal driving input. The wing pitch kinematics (blue) contain higher harmonic components because its motion is passive.

In this underactuated flapping-wing system, the wing stroke and pitch motion are strongly coupled. An increase in wing pitch amplitude $\left(\psi \right)$ corresponds to a decrease in net drag force [25], which leads to an increase in the wing stroke amplitude (ϕ). In this robot design, the flapping kinematics of the hindwings are strongly influenced by the downwash induced by the forewings. This downwash changes the hindwings’ passive pitch motions, which further changes their stroke amplitudes. Compared to the in-phase flapping test, the robot hindwings’ stroke amplitudes increase by 7° in the out-of-phase experiment. This observation implies that varying the phase θ can lead to a noticeable change of the flapping kinematics, which further influences lift force production.

**Figure 6:**
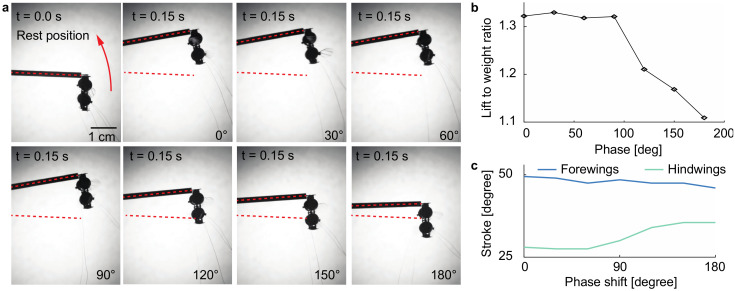
Liftoff experiments for quantifying the influence of phase shift θ on lift force production. (**a**) Robot rest position and final positions. In these experiments, the robot is driven at 1550 V, 350 Hz for 0.15 s while varying the phase shift θ. (**b**) Measured robot lift-to-weight ratio as a function of phase shift θ. (**c**) Measured forewing and hindwing stroke amplitudes as functions of phase shift θ. The stroke amplitudes shown in (**c**) are the average values of the left and right wings.

To quantify the influence of phase shift θ on lift force generation, we perform liftoff experiments using the setup described in Section 2.2.2. We drive the robot at 1550 V, 350 Hz, and vary the phase shift θ from 0° to 180° in steps of 30°. Figure 6a shows the robot rest position and the final positions for these liftoff experiments. For each liftoff experiment, we measure the final rotation angle $\gamma_{f}$ and then use Equation (4) to calculate the mean lift force. Figure 6b shows the robot lift force as a function of phase shift θ. The robot lift-to-weight ratio is 1.32 when the phase shift θ is smaller than or equal to 90°. The lift-to-weight ratio sharply decreases when the phase shift θ exceeds 90°. When the forewing and hindwing stroke motion becomes out-of-phase (θ=180°), the robot lift-to-weight ratio decreases to 1.11. This observation shows that varying θ can lead to a 19% change in net lift production. Supplementary Video S2 compares in-phase and out-of-phase liftoff experiments.

To analyze this observation, we drive the robot at the same operating conditions and measure its stroke amplitude as the phase shift θ changes. Figure 6c compares the average (of left and right wings) forewing and hindwing stroke amplitude as a function of θ. In the out-of-phase experiment, the hindwing stroke amplitude increases by 7° while the forewing stroke amplitude decreases by 3°. We believe this measurement implies that out-of-phase flapping induces a weaker downwash. The hindwings have larger stroke and pitch motion, which implies they are less impacted by the downwash from the forewings. Per momentum theory, under constant power, both lift force and stroke amplitude are influenced by the downwash. This kinematics measurement supports the prior observation that out-of-phase flapping reduces lift force generation.

### 3.3. Influence of Driving Frequency f on Lift Force Production and Stroke Amplitudes

**Figure 7:**
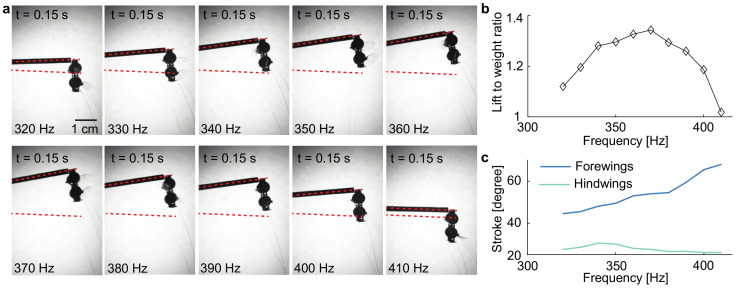
Effect of driving frequency f on lift force production. (**a**) Final robot liftoff positions at frequencies ranging from 320 Hz to 410 Hz. (**b**) Robot lift-to-weight ratio as a function of driving frequency. (**c**) Forewing and hindwing stroke amplitudes as a function of driving frequency.

In addition to investigating the influence of phase shift θ on lift force production, we also vary the driving frequency f in the range of 320 Hz to 410 Hz. Figure 7a compares the liftoff positions as a function of f. Based on the measured rotation angle $\gamma_{f}$ in Figure 7a, we calculate the robot lift-to-weight ratio. Figure 7b shows that the robot lift peaks at 370 Hz. From 340 to 390 Hz, the robot lift varies by less than 10%. Compared to state-of-the-art sub-gram flapping-wing robots powered by piezoelectric actuators [6], this robot’s resonance peak is approximately ten times wider. This observation implies two robot properties: 1) this robot is less efficient than piezoelectric-driven robots owing to its lower quality factor; and 2) this robot is easier to operate because varying operating frequencies in a large range (340 to 390 Hz) does not severely impact lift force production.

We further measure the forewing and hindwing stroke amplitudes as functions of the driving frequency f. Figure 7c shows that the forewings’ average stroke amplitude increases as the driving frequency increases. However, a monotonic increase in the forewing’s stroke amplitude does not correspond to lift increase at higher frequencies.

### 3.4. Influence of Voltage Amplitude $V_{amp}$ on Lift Force Production and Stroke Amplitudes

**Figure 8:**
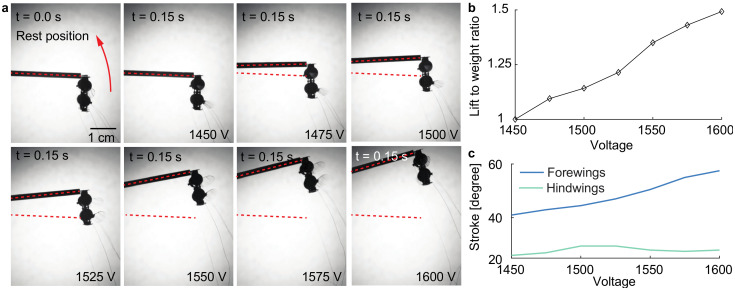
Effect of driving amplitude $V_{amp}$ on lift force production. (**a**) Final robot liftoff positions at driving frequencies ranging from 1450 V to 1600 V. (**b**) Robot lift-to-weight ratio as a function of driving voltage amplitude. (**c**) Forewing and hindwing stroke amplitudes as a function of $V_{amp}$.

We further investigate the influence of driving voltage $V_{amp}$ on lift force production. Figure 8a compares the robot liftoff performance as we change the driving voltage from 1450 V to 1600 V. Based on the measured rotation angle $\gamma_{f}$, we calculate the robot lift-to-weight-ratio as a function of $V_{amp}$. Figure 8b shows that the lift force increases monotonically as the driving voltage increases. The robot’s maximum lift-to-weight-ratio is 1.49 at the operating condition of 1600 V, 350 Hz, and 0° phase shift.

We also measure the forewing and hindwing stroke amplitudes at these driving conditions. Figure 8c shows that the forewings’ average stroke amplitude increases at higher driving voltages. Unlike experiments shown in Figure 7, the increase in stroke amplitude of the forewings here leads to a monotonic rise in the lift.

## 4. Discussion

This set of at-scale experiments confirms the findings in previous computational and biological studies [16]. Prior studies found that dragonflies use in-phase flapping to augment lift during takeoff and switch to out-of-phase flapping while hovering to save energy and improve stability. Our main measurements in Figure 6 confirm that in-phase flapping augments lift production. However, our design cannot quantify the effect of phase shift on aerodynamic efficiency. Despite producing different lift forces, in-phase and out-of-phase flapping consume similar amounts of electrical power. In both experiments, the DEAs consume a net power of 0.4 W because the electrical dissipation is substantially larger than aerodynamic loss. Hence, for our robot, it is advantageous to flap forewings and hindwings in-phase as this strategy increases net lift without requiring additional power. To reduce power consumption, future works need to improve the DEA transduction efficiency. This requires a reduction of DEA electrode resistance and elastomer viscoelasticity [14].

### Stroke Amplitudes, Lift Production, and Wing Pitch

In Section 3.3 and Section 3.4, we found that increasing the driving frequency f and voltage amplitude $V_{amp}$ similarly results in the stroke amplitude of the forewings. However, the enlarged stroke amplitudes do not immediately lead to an increase in lift in the former case. This is likely related to the wing pitch kinematics.

As observed in Figure 7, the lift peaked when the flapping frequency reached 370 Hz, despite the continuing growth in stroke amplitude of the forewings at higher frequencies. This is likely because, beyond 370 Hz, the wing pitch amplitude grows beyond 45° and the stroke-pitch phase shift becomes positive. As shown in our prior work [25], delayed wing pitch rotation leads to a sharp reduction in lift force.

On the other hand, as manifested by Figure 7, the increase in the driving voltage does not lead to change of stroke-pitch phase shift [25]. Consequently, the net lift force increases due to the stroke amplitude increase.

Furthermore, in both scenarios, we observe that the hindwings do not experience an increase in stroke amplitude. This could be caused by the downwash generated through forewing–hindwing interactions. However, if the forewings are switched off, then the hindwing amplitude grows to 36°. This observation suggests that the induced downwash has a significant effect on the hindwing flapping kinematics. Future studies should quantify this this interaction by measuring the induced flow. Performing at-scale particle image velocimetry (PIV) experiments to measure the induced flow could be a part of future work in addition to an attempt to optimize hindwing shape, inertia, and hinge stiffness.

## 5. Conclusions

In this paper, we developed a 317 mg soft robotic dragonfly powered by two DEAs. We measured the robot flapping kinematics and the associated lift forces at different driving voltages, frequencies, and relative phases. The robot achieved a maximum lift-to-weight ratio of 1.49 to 1 while consuming 0.4 W of electrical power. We further demonstrated that in-phase flapping leads to a 19% lift force increase compared to out-of-phase flapping. This work presents the first at-scale dragonfly-like robot capable of liftoff, showing the promise of a novel bio-inspired design.

This robotic platform could lead to several future research studies. First, our current robot uses identical forewing and hindwing designs. In our experiments, we observe that strong forewing–hindwing interactions can influence the hindwing pitching dynamics, which could further impact lift force production. Future studies should quantify the wing–wing interactions through measuring the induced downwash [24]. Based on the measurement, future studies should optimize hindwing designs, adjust hindwing stiffness, and potentially change the hindwing transmission ratios. These tasks could substantially improve hindwing performance and efficiency. To complement the experimental studies, future computational investigation of inertial and viscous forces [26,27] in the intermediate Reynolds number regime can also benefit wing, wing hinge, and transmission optimization.

Second, prior computational and biological studies [20] showed that dragonflies have tilted wing stroke planes. A dragonfly leverages this tilted stroke plane and drag-based forces to enhance net lift force production. In this work, we simplified the actuation design to having a horizontal stroke plane. Future work could experiment with tilted stroke plane designs and investigate the complex mechanisms of forewing–hindwing interactions.

Finally, this work was limited to presenting robot flapping kinematics and liftoff measurements. To demonstrate controlled hovering flight, future work should derive the system dynamical model and design a feedback controller. While in-phase flapping augments lift force, it can cause large body oscillations. Similar to natural dragonflies, the robot may alternate between in-phase and out-of-phase flapping during takeoff and hovering flight.

## Supplementary Materials

The following supporting information can be downloaded at: https://www.mdpi.com/article/10.3390/mi13071136/s1, Video S1: High-speed video of a static flapping-wing experiment; Video S2: High-speed video of a robot liftoff experiment.
